# Association between grip and core muscle strength in people with axial spondyloarthritis and healthy controls

**DOI:** 10.1186/s41927-025-00575-y

**Published:** 2025-10-21

**Authors:** Anne-Kathrin Rausch Osthoff, Marina Bruderer-Hofstetter, Lea Ettlin, Fiona Bischofberger, Selina Papritz, Alexandra Schwab, Franco Weidmann, Karin Niedermann

**Affiliations:** 1 Institut of Physiotherapy, School of Health Sciences, ZHAW Zurich University of Applied Sciences, Katharina-Sulzer-Platz 9, Winterthur, 8401 Switzerland; 2 SVMB, Ankylosing Spondylitis Associations Switzerland, Leutschenbachstrasse 45, Zurich, 8050 Switzerland; 3 Division of Physiotherapy, School of Health Professions, Bern University of Applied Sciences, Murtenstrasse 10, Bern, 3012 Switzerland; 4 Institut of Public Health, ZHAW Zurich University of Applied Sciences, Katharina-Sulzer-Platz 9, Winterthur, 8401 Switzerland

**Keywords:** axSpA, Assessments, Exercise, Strength

## Abstract

**Background:**

Annual fitness assessments are performed during group exercise therapy for people with axial Spondyloarthritis (axSpA) living in Switzerland. The core strength test (CST) is time-consuming, and interpretation limited. Thus, the objectives were to 1) compare the CST-performance of people with axSpA and healthy controls, and 2) evaluate if hand grip strength can be used as a proxy for core strength.

**Methods:**

Routinely gathered data of people with axSpA was used and data from healthy controls collected. Differences in strength were investigated using Welch Two-sample t-tests or Fisher’s exact tests. The associations between grip and core strength were explored through pairwise Pearson correlations (r). Further, a linear regression model was fitted to data of people with axSpA with grip strength as the response variable, and ventral, dorsal and lateral core strength endurance, age and sex as predictors.

**Results:**

Data from 160 healthy controls (50% male, mean age 59.3 (SD 11.47) years) and 112 people with axSpA (58% male, mean age 57.7 (SD 12.1) years) was included. People with axSpA showed lower core strength endurance (sec) than the controls: ventral core strength mean difference −28, *p* < 0.001; lateral core strength mean difference −17, *p* < 0.001; dorsal core strength mean difference −39, *p* < 0.001, and lower grip strength −3.7, *p* = 0.012. The linear regression model with hand grip as response and core strength, age, and sex as predictors explained 44% of the variability.

**Conclusion:**

People with axSpA showed substantially lower core muscle strength endurance than healthy controls. Core strength measures have only marginal effects on grip strength in people with axSpA. Therefore, grip strength is not appropriate to be used a s a proxy for core strength in people with axSpA and healthy people.

**Clinical trial number:**

Not applicable.

**Supplementary information:**

The online version contains supplementary material available at 10.1186/s41927-025-00575-y.

## Key points

• People with axial spondyloarthritis show lower core strength endurance than healthy controls

• Grip strength is not appropriate as a proxy for core strength in people with axial spondyloarthritis nor healthy individuals.

## Background

Axial Spondyloarthritis (axSpA) is a chronic inflammatory rheumatic condition mainly affecting the axial skeleton, but also peripheral and musculoskeletal manifestations are frequently observed [1]. Individuals with axSpA present structural and functional impairments, as well as comorbidities, impacting the quality of life, working ability and physical fitness [2–5].

Disease management guidelines recommend the combination of pharmacological and non-pharmacological modalities [1]. The first-line treatment consists of non-pharmacological treatment which is based on education and physical activity [1]. Based on the public health guidelines for physical activity by the World Health Organisation [6], the regular performance of aerobic, muscle strength, flexibility and neuromotor exercises is recommended [7]. The 2018 EULAR recommendations for physical activity in people with inflammatory arthritis and osteoarthritis underpin the relevance of exercise in people with axSpA, given its effectiveness, feasibility and safety [7].

Currently, the Swiss Ankylosing Spondylitis Association (Schweizerische Vereinigung Morbus Bechterew, SVMB) has more than 4300 members and runs more than 60 exercise groups across Switzerland. Consistently to the 2018 EULAR recommendations for physical activity, the SVMB offers several delivery modalities promoting physical activity and exercise, embedded in groups, events or in individual settings. Both concepts, called BeFit (groups) and myBeFit (individual settings), include, among others, regular fitness assessments [8]. These are used for defining and evaluating individual exercise goals. According to the four domains of exercises [9], the assessment test battery includes one test for each domain. Core muscle strength is measured using the Core Strength Endurance test battery (CST), which was evaluated as a reliable and feasible assessment in people with axSpA. [10]. During the performance of this test, two challenges are faced: since no norm data of health controls aged 40 or older are available, the interpretation of results is only possible in an intra-individual way. However, participants with axSpA wish to compare their test results with peers and healthy controls. Furthermore, the CST is time-consuming, which significantly impacts the overall amount of time required for testing [8]. Hence, we are seeking for an established test, low in costs, which can be easily applied in different clinical settings, particularly in the diverse groups therapy settings, e.g. gym, swimming pool, physiotherapy/rehabilitation clinic, exercise room, community room. One option is hand grip strength test, which needs only little time and is already used as a substitute for multiple fitness-related tests [11]. Even though literature is inconclusive whether or not hand force is suitable as an indirect measuring instrument for core and lower limb force [12], there is evidence showing that a strong core is associated with better grip-based performance in health individuals [13, 14].

Accordingly, the study aims to: 1) compare the CST-performance of people with axSpA and healthy controls, and 2) to evaluate if hand grip strength measurement can be used as a proxy for core strength endurance to reduce the assessment time in people with axSpA.

## Methods

### Study design

A prospective cross-sectional study was conducted gathering data in healthy people. Furthermore, routinely gathered data of people with axSpA were retrospectively used for analysis. The findings are reported in line with the STROBE statement for cross-sectional studies [15]. A registration of the study was not required (Clinical trial number: not applicable).

The study obtained ethical approval from the Ethics committee Canton Zurich (BASEC no. 2018–00145, adjustment July 2020) and all participants gave written informed consent. The study was conducted in accordance with the Declaration if Helsinki [16]. The collected data were anonymized.

## Participants

### Healthy people

Healthy people between 40 and 79 years of age were recruited from local sports clubs and university staff. For each age decade twenty women and men were included. The inclusion criteria were age > 18 years, German-speaking, subjective well-being, and the ability to perform the quadrupled stance, side plank and prone position on the floor. Exclusion criteria were back surgery in the last three months, competitive athletes who exercise more than five times a week, and medical conditions, limiting range of joint movement required for the tests.

Health was defined by the World Health Organisation (WHO) in New York in 1946 as followed: ‘Health is a state of complete physical, mental and social well-being and not merely the absence of disease or infirmity.’ [17]. Being healthy is therefore a personal perception and was assessed individually by the participants. The question evaluating the state of health used was ‘Do you feel healthy?’ or ‘Do you feel fully functional and not restricted in everyday activities and participation in social life despite diagnosed diseases?’. Answering both questions with ‘yes’ meant the presence of a subjective state of health and therefore inclusion in the study.

All participants gave written informed consent and completed a screening questionnaire, afterwards the measurements were scheduled. The following data were collected for participants characteristics: sex, age, body weight, height and smoking status.

### People with axSpA

Data of people with axSpA were retrospectively taken from the SVMB database of routinely annually gathered assessment data [8]. The extracted data included demographics, disease activity, years since disease onset, core strength, and hand grip strength.

## Procedure in healthy people

The assessments of the healthy people were carried out by students of the bachelor’s degree programme in physiotherapy at the university and supervised by a senior physiotherapist at their group exercise settings across Switzerland. The entire assessment procedure and the correct instruction were standardized and trained in advance (2 hours workshop, supervision during assessments by senior assessor). Verbal motivation to the participant was not allowed. Before starting the assessments, the participants were explicitly instructed to exert themselves to the maximum while performing the assessments. The breaks between the individual exercises were two minutes.

## Assessments

### Health status in people with axSpA

The health status was described using two established axSpA-specific quuestionnaires:

The Bath Ankylosing Spondylitis Disease Activity Index (BASDAI) is a self-reported questionnaire consisting of 6 items related to the major symptoms fatigue, pain, stiffness, swelling, tenderness which are rated by use of a 0 (no problem) −10 (worst problem) scale [18]. A Score < 4 indicates a suboptimal disease control. The German version is reliable and sensitive to change [19].

The ADAS Health Index (ASAS-HI) is a self-reported questionnaire consisting of 17 items covering pain, emotional functions, sleep, sexual function, mobility, self-care, and community life [20]. The answer options are dichotomous, the lower the score the better the health status. The ASAS-HI German version is reliable and validated for its use in the Swiss population [21, 22].

### Core strength

**Fig. 1:**
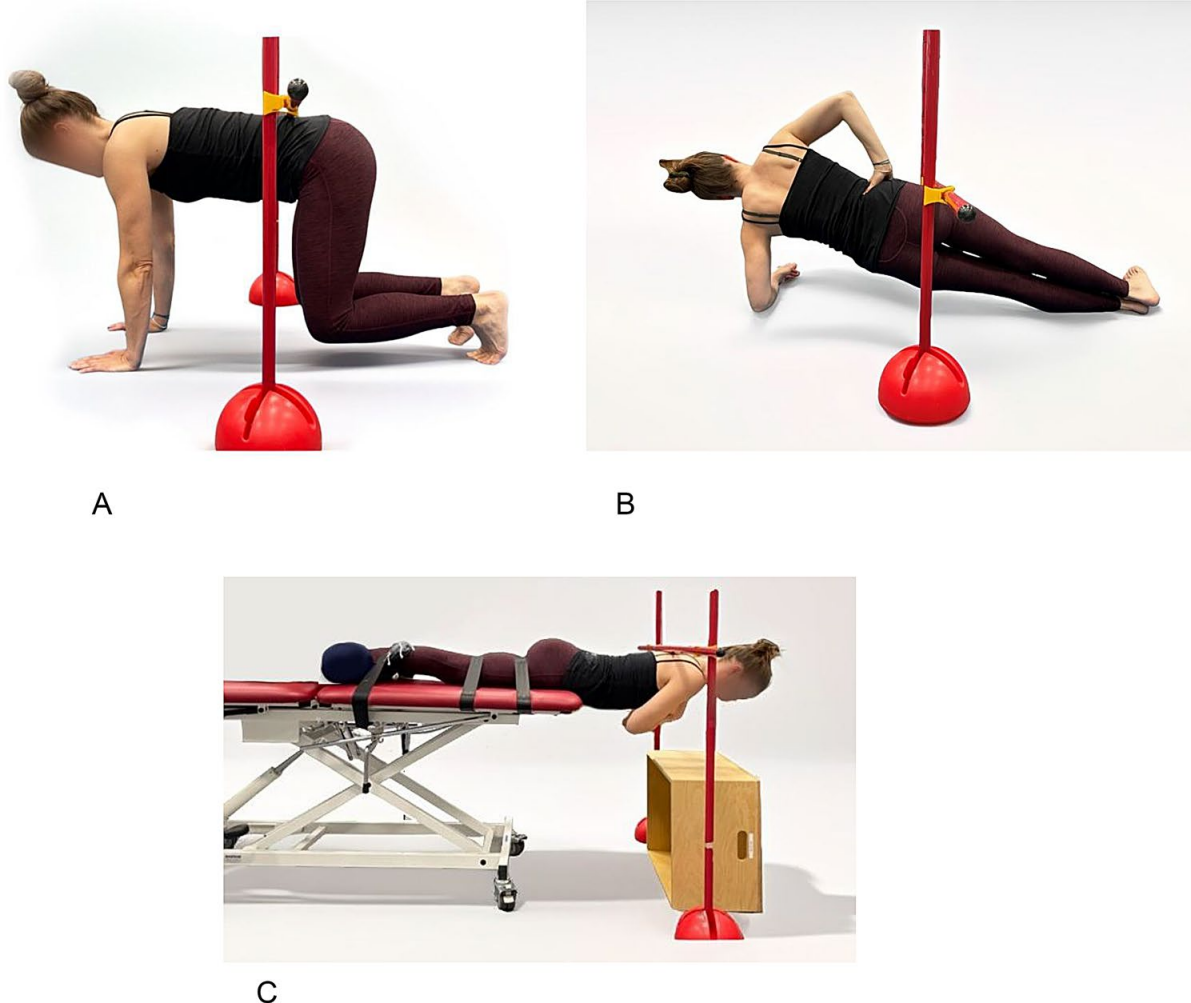
(**A**): Ventral plane of the CST. (**B**): Lateral plane of the CST. (**C**): Dorsal plane of the CST

The CST was originally developed by the Swiss Olympic Medical Center for the use with athletes and designed to evaluate the “basic” core strength, meaning the minimum strength required for the performance of sports [23, 24]. The adapted version of the CST measures the isometric strength of the ventral, lateral and dorsal muscle chains in people with axSpA showing a moderate to substantial intra-rater reliability [10]. Participants are asked to hold the position in Fig. 1A (ventral plane), 1B (lateral plane), 1C (dorsal plane) as long as possible, the time is measured in seconds.

### Grip strength

Grip strength was measured using a hand dynamometer (Jamar® hydraulic hand dynamometer). This is one of the most common instruments assessing grip strength and is characterized by its ease of use, validity and reliability [25]. The participant sat on a chair with a back- but no armrests. The back was touching the backrest and the feet were placed hip-distance apart. The upper arm was in contact with the rib cage, the flexion of the elbow 90° [26], the wrist in 0–30° dorsal extension [27]. As recommended by Trampisch et al. [28], the grip was set to position 2 for all participants and each hand was measured three times in turns without predefined rest-intervals. The results were recorded in kilogram force [kgf]. The mean of the self-reported dominant hand was calculated and used for all analyses.

## Statistical analysis

All analyses were performed using the R statistical software R version 4.3.2 [29]. To investigate differences between both groups, relevant demographics, core strength endurance, and grip strength measures were compared using Welch Two-sample t-tests and Fisher’s exact tests, if appropriate. The alpha level was adjusted by the Bonferroni method. The associations between grip strength and core strength endurance measures were explored through pairwise Pearson correlations (r) in both groups, as well as sex-specific subgroups. In addition, we fitted a linear regression model with axSpA using the lm function from the base package. The estimates were summarized using the tbl_regression function from the gt_summary package [30]. Model diagnostics included visual inspection of the diagnostic plots using the check_model function from the performance package [31], including linearity of the data, normality of the residuals homogeneity of variance, multicollinearity, influential observations, posterior predictive check. In addition, the Breusch-Pegan test was used to test for heterogeneity, the variance inflation factor (VIF) and Durbin-Watson Test were used to check for multicollinearity using the lmtest package version 09–40 [32] within R.

## Results

Data from both groups was collected between April 2021 and December 2022. Data from 112 people with axSpA and 160 healthy people were included (Table 1). In people with axSpA, mean years since symptom onset were 37.3 (SD 24.9), and since diagnosis 29.4 (SD 27.1), based on fifty observations. The median BASDAI score was 2.5 (IQR 1.7–4.2, *n* = 18) and the median ADAS Health index was 3 (IQR 0–7, *n* = 55). Demographic characteristics (i.e., age, sex, weight, height, BMI, and smoking status) did not differ between the two groups. However, people with axSpA showed less ventral, dorsal, and lateral core strength endurance (Table 1).

**Table 1:** Demographic and clinical characteristics

Demographics	People with axSpA (*n* = 112)	Healthy (*n* = 160)	*Between group differences (95% CI)
Age (mean, SD)	57.7,12.09	59.3,11.47	1.59 (−4.47–1.28)
range	23–81	40–79	
Female sex (%)	47 (42%)	80 (50%)	0.72 (0.43–1.2)
Height in cm (mean, SD)	172.4, 8.35	171.3, 8.97	1.15 (−0.96–3.26)
range	154–197	148–191	
Weight in kg (mean, SD)	72.4, 13.87	70.6, 11.89	1.75 (−1.46–4.98)
range	45–113	48–108	
BMI (mean, SD)	24.3, 4.24	24, 3.13	0.31 (−063–1.25)
Range	17.3–36.9	18.1–36.9	
Smoking status yes (%)^1^	9 (8.3%)	13 (8%)	0.97 (0.37–2.68)
**Strength measures**			
Grip strength in kgf^2^ (mean, SD)	33, 10.27	36.7, 13.55	−3.7 (−6.5 – −0.81)
range	13–60.7	15–80.7	t = −2.5268, *p* = 0.012
Core strength endurance			
Ventral in seconds^3^ (mean, SD)	69.6, 40.8	97.6, 57.13	−28 (−40 – −16)
range	3–195	11–36	t = −4.69, *p* < 0.001
Dorsal in seconds^4^ (mean, SD)	67.1, 47.82	105.6, 53.36	−39 (- 26 – −51)
range	0–245	8–285	t = −6.06, *p* < 0.001
Lateral in seconds^5^ (mean, SD)	39.4, 24.69	56.6, 33.63	−17 (−24 – −10)
range	5–140	4–197	t = −4.81, *p* < 0.001

*Between group differences based on differences in means for continuous variables and based on odds ratios for categorical variables

Abbreviations: SD, standard deviation; cm, centimeter; kg, kilogram; kgf, kilogram force

^1^n in axSpA = 108, ^2^ n in axSpA = 110, ^3^ n in axSpA = 109, ^4^ n in axSpA = 101, ^5^n in axSpA = 109

Descriptives on the strength measures for people with axSpA and healthy people overall and per age-group are summarized in Table 2. In both groups in male participants and younger age decades higher mean strength measures were observed, however with wide ranges.

**Table 2:** Sex and age specific core strength endurance and grip strength measures in people with axSpA and healthy people

		People with axSpA		Healthy people	
		female	male	female	male
	Age decade*				
**Strength measures**					
Grip strength in kilogram force					
mean (SD)	30–39 yrs	25.5 (4.5)	45.9 (10.3)	n/a	n/a
median (range)		25.5 (22.3 - 28.7)	45.3 (36.0 - 60.0)		
	40–49 yrs	24.9 (3.9)	41.2 (9.9)	31 (4.9)	55.9 (14.2)
		25 (20.3 - 32.3)	43.3 (28 - 50.7)	31 (20.0 - 39.7)	58.2 (30 - 80.7)
	50–59 yrs	29.6 (8.6)	39.2 (9.0)	30.3 (5.5)	48.3 (7.3)
		29.3 (16.7 - 47.3)	40.4 (22.7 - 53.3)	30.0 (19.0 - 39.3)	48.4 (33.3 - 62.0)
	60–69 yrs	24.6 (6.8)	41.0 (6.5)	25.2 (5.8)	41.8 (8.3)
		24 (13.0 - 34.7)	39.3 (27.3 - 52)	24.0 (16.3 - 36.7)	39 (30.0 - 62.0)
	70–79 yrs	22 (1.8)	30.8 (9.0)	21.4 (3.9)	39.4 (9.1)
		22 (20.7 - 23.3)	30.5 (17.0 - 50.7)	20.6 (15.0 - 30.0)	41.5 (18.3 - 52.0)
**Core strength endurance in seconds**					
Ventral					
mean (SD)	30–39 yrs	51.5 (19.1)	86.6 (34.6)	n/a	n/a
median (range)		51.5 (38 - 65)	82 (38 - 131)		
	40–49 yrs	74.2 (57.7)	76 (46.3)	88.4 (34.2)	121.9 (56.9)
		55 (14 - 195)	68 (30 - 128)	84 (35 - 175)	106 (51 - 255)
	50–59 yrs	60.1 (34.5)	89.3 (35.6)	83.1 (27.7)	135 (55.1)
		52 (14 - 138)	100 (41 - 153)	80 (42 - 136)	131 (61 - 231)
	60–69 yrs	69.3 (49.7)	70.7 (43.0)	96 (91)	99 (44.9)
		59.5 (3 - 188)	61 (16 - 149)	62 (25 - 361)	94 (19 - 197)
	70–79 yrs	51 (5.7)	57.7 (36.3)	61.6 (42.9)	94.9 (56.2)
		51 (47 - 55)	59 (8 - 137)	56 (11 - 183)	86 (11 - 232)
Dorsal					
mean (SD)	30–39 yrs	49.5 (33.2)	60.8 (25.9)	n/a	n/a
median (range)		49.5 (26 - 73)	63 (33 - 97)		
	40–49 yrs	72.3 (38.2)	63.5 (41.6)	137 (54.3)	124 (48.8)
		62 (16 - 128)	58.5 (22 - 115)	118 (60 - 268)	104 (56 - 219)
	50–59 yrs	71.1 (42.5)	65.7 (50.2)	127 (51.1)	106 (32.6)
		63 (29 - 192)	56 (9 - 170)	122 (55 - 235)	103 (66 - 167)
	60–69 yrs	77.8 (48.1)	61.4 (44.6)	98.6 (66)	92.2 (40.4)
		75 (8 - 145)	52 (0 - 159)	92 (26 - 285)	82 (27 - 182)
	70–79 yrs	62.5 (14.8)	56.2 (70.3)	74.1 (53.6)	86.2 (49.1)
		62.5 (52 - 73)	41 (0 - 245)	56.5 (8 - 205)	100 (8 - 185)
Lateral					
mean (SD)	30–39 yrs	35 (0)**	57.4 (31)	n/a	n/a
median (range)		35 (35 - 35)	61 (15 - 91)		
	40–49 yrs	29.6 (19.9)	46.4 (36.6)	60.6 (20.4)	75.4 (20.9)
		23 (6 - 76)	32 (12 - 105)	57.5 (23 - 116)	67.5 (44 - 126)
	50–59 yrs	32.2 (15.4)	53.5 (20.7)	47.4 (27.1)	84.1 (33.4)
		33 (6 - 63)	47 (25 - 108)	42.5 (9 - 102)	72 (35 - 153)
	60–69 yrs	30.4 (23.8)	43.5 (20.0)	41.6 (38.2)	67.8 (44.1)
		28.5 (5 - 109)	41 (17 - 71)	35.5 (8 - 156)	59.5 (20 - 197)
	70–79 yrs	23.5 (0.7)	32.6 (22.4)	28.6 (18.3)	46.9 (21.2)
		23.5 (23 - 24)	28 (5 - 90)	28.5 (4 - 67)	46.5 (16 - 95)

*Age decade in healthy people based on 20 observations per decade and sex in people with axSpA based on 7 (30–39 years), 14 (40–49 years), 31 (50–59), 33 (60–69), and 22 (70–79) observations, data from four individuals age < 30 and one individual > 80 were not considered

**only two females were included in this decade with the same test result

Abbreviations: SD, standard deviation; yrs, years ; n/a, not applicable

**Fig. 2:**
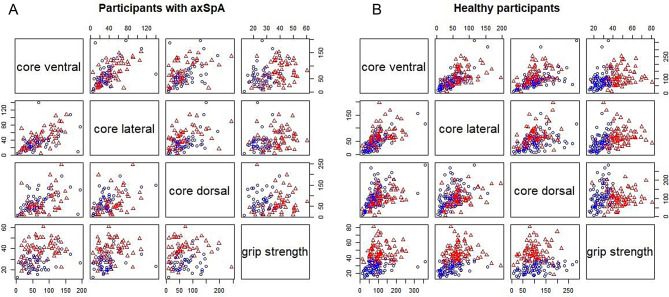
Pairwise scatterplots in people with axSpA (**A**) and healthy people (**B**). Blue circles represent female participants, and red triangles male participants

Pairwise correlations based on complete cases in the people with axSpA and healthy people and sex-specific subgroups are summarized in Table 3, pairwise scatterplots are presented in Fig. 2. Correlations ranged between *r* = 0.07 between dorsal muscle endurance and grip strength in healthy men to *r* = 0.44 between lateral muscle strength endurance and grip strength in healthy women.

**Table 3:** Pairwise correlations between hand grip strength and core muscle strength endurance measures in people with axSpA and healthy people in sex specific subgroups

	People with axSpA (*n* = 99)*			Healthy people (*n* = 160)		
		females (*n* = 43*)	males (*n* = 56*)		females (*n* = 80)	males (*n* = 80)
Ventral	r = 0.34	r = 0.31	r = 0.31	r = 0.31	r = 0.31	r = 0.12
(95% CI)	(0.14 to 0.49)	(0.02 to 0.56)	(0.06 to 0.52)	(0.17 to 0.45)	(0.09 to 0.49)	(−0.1 to 0.33)
Lateral	r = 0.28	r = 0.2	r = 0.21	r = 0.38	r = 0.44	r = 0.09
(95% CI)	(0.09 to 0.44)	(−0.1 to 0.47)	(−0.04 to 0.44)	(0.24 to 0.51)	(0.25 to 0.6)	(−0.13 to 0.3)
Dorsal	r = 0.05	r = 0.19	r = 0.15	r = 0.08	r = 0.41	r = 0.07
(95% CI)	(−0.15 to 0.24)	(−0.11 to 0.47)	(−0.12 to 0.39)	(−0.07 to 0.24)	(0.21 to 0.58)	(−0.16 to 0.28)

*Pairwise correlations on complete cases

Abbreviations: r, Pearson correlation coefficient; CI, 95% Confidence Interval

We fitted a linear regression model with grip strength as the response variable, ventral, dorsal and lateral core strength endurance, age and sex as predictors. Pairwise scatterplots were established to explore a linear relationship between grip strength and core muscle endurance measures (Figs. 2A and 2B). The model explained 44% of the variance. The residual standard deviation was 8.06 on 93 DF, the R^2^ = 0.44 (adjusted R^2^ = 0.41) and F-statistic = 14.77 on 5 and 93 DF (*p* < 0.001). Sex and age were the predictors with the most considerable effects, and the only significant effects after adjusting for multiple testing. All estimates are summarized in Table 4. Visual inspection of the diagnostic plots indicated no evidence of a violation of model assumptions (Appendix A). The Breusch-Pegan test did not indicate the presence of heteroscedasticity (BP = 4.3086, DF = 5, *p* = 0.5059), and the Durbin-Watson test (DW = 2.0311, *p* = 0.5406) and VIF (ranging from VIF = 1.25 age to VIF = 2.13 lateral core strength endurance) did not indicate multicollinearity.

**Table 4:** Effect estimates based on linear regression on grip strength in people with axSpA

Characteristic	Estimate	95% CI	t-value	p-value	q-value*
Intercept	36	26 to 46	7.172	< 0.001	< 0.001
Ventral	0.06	−0.03 to 0.05	2.1	0.03856	0.2
Lateral	−0.04	−0.13 to 0.05	−0.84	0.40300	> 0.9
Dorsal	−0.01	−0.03 to 0.05	0.44	0.65798	> 0.9
Age	−0.24	−0.39 to −0.08	−2.95	0.00402	0.024
Sex (male)	13	9.5 to 17	6.96	< 0.001	< 0.001

Abbreviations: CI, Confidence interval

*Bonferroni correction for multiple testing

## Discussion

Our study showed that people with axSpA have lower hand grip strength and core strength endurance than healthy controls. This finding is not surprising and in line with other studies showing that the general body muscle strength of people with axSpA is lower than in healthy controls [33–35]. It has been suggested that strength in people with axSpA can be limited by several symptoms caused by the chronic inflammation, expressed as pain or fatigue, and functional impairments (e.g. reduced range of motion) leading to reduced test tolerance [5, 34]. Additionally, reduced muscle strength is associated with impairments in self-reported physical functioning and performance tests e.g. gait speed [36].

Several studies confirmed that people with aSpA show lower core strength than healthy controls using different devices and approaches, such es hand-held-dynamometer [33], isokinetic device [34], or time measures [37]. There is no consensus on a gold standard for the measurement of core strength available. Core strength is a relevant premise for core stability, which is generally discussed having the components endurance, motor control, function and strength [38], however, core stability has no clear definition yet [39, 40]. The initiative ASAS OMERACT defined core domain sets for assessments in studies with people living with axSpA, however, no recommendations for the assessment of strength was provided yet unless the Bath Ankylosing Functional Index which is a proxy for function in daily activities [41, 42]. Still, measuring strength serves as fundamental metric of muscle function and overall physical capacity. Particularly pertinent to the aging population, handgrip strength is closely associated with sarcopenia, which is characterized by the progressive and generalized loss of skeletal muscle mass, strength, and functional capacity that occurs with advancing age. Therefore, hand grip strength was suggested to a vital sign of health [43].

The measurement provided data that will be useful in interpreting the core strength performance development of each individual with axSpA participating in the annual (my)BeFit assessments. The individual test results can be compared with the results of previous years as well as with data of comparable peers, e.g. same age or healthy controls. Even though normative values are usually based on a higher sample size [44, 45], this study allows a helpful interpretation. This information is a useful base for individual exercise promotion according to the 2018 EULAR recommendation for the management of rheumatic diseases [1, 7], including physical activity counselling and assessments as implemented by the SVMB. Regular fitness assessments should be part of standard care and screen for all fitness dimensions with potential of improvements and efficient planning of the training, as well as predictor for disease activity. Additional to strength and mobility, which can be seen as the fitness dimensions of ‘traditional focus’ in the treatment of axSpA, cardiovascular fitness is now considered very relevant for people with axSpA as they have an increased risk for cardiovascular diseases [46, 47]. There is evidence showing that cardiovascular training has positive impact on disease activity and reduces the risk for cardiovascular diseases [48]. Therefore, assessments and training should include the cardiovascular fitness dimension [49, 50].

World Physio underpins the necessity of the standard use of assessments [51]. A standard assessment needs to show good psychometric properties as well as high applicability. Therefore, the assessment needs to be feasible in different settings such as small physiotherapy practices, sport facilities or large rehabilitation centers as well as individual or group therapy. Accordingly, assessments need to be easy to perform and low in cost and time requirements [52]. Even though physiotherapists have a generally positive attitude towards the use of assessments, physiotherapists in the German-speaking countries Austria, Germany and Switzerland report a number of barriers for using them in daily routine, such as lack of financial compensation, time expenditure, or complex instructions [53–56]. Having this lack of resources in mind, the secondary aim of this study was to investigate if the hand grip strength test can substitute the more complex procedure of the CST. The results of this study debunked this hypothesis. In future, it might be reasonable to search for other peripheral muscle strength assessments easy and quick in performance as a substitute for the CST, e.g. quadriceps strength.

## Limitations

In people with axSpA, data from the regular fitness assessment were analyzed retrospectively. Although these measurement procedures were standardized and performed by trained staff, the comparability with the data of healthy people may be limited due to different raters and settings. In addition, the quality of the data in a database is highly dependent on the data entry. Four cases had to be excluded from the analysis due to probable errors during data entry. Another problem was the different handling of missings, as the reason for the missings was unclear, e.g. was the person unable to perform a test or was the test not carried out. In addition, information on the mean years since symptom onset and since diagnosis, BASDAI score and ADAS Health index was not available for all participants with axSpA, therefore, the health status of the entire group can only be described to a limited extent. Therefore, caution is advised when transferring the results of core strength and grip strength measures to people with axSpA. Moreover, due to the incomplete data from persons with axSpA, it was not possible to include other potentially confounding variables, e.g. smoking status or disease severity, in the regression analysis. Furthermore, we did not match healthy people and people with axSpA for gender or age, which could affect the validity of the group comparisons. Age and sex itself have an impact on muscle strength and function [57]. Finally, because our study was explorative in nature no a priori sample size calculation was conducted. The total number of observations in each cell per age and sex group are too low to conclude on normative values. The low numbers may have resulted in imprecise estimations for means and standard deviations. In addition, for some variables we observed skewed dispersions. Therefore, our results have to be treated with caution and in order to develop norm data for core strength endurance, the number of included persons needs to be increased in future studies.

Two specific tests were used to evaluate strength. Hand grip strength was tested by three maximal contractions and condensed by the mean of all three measures. This analysis is accepted and known as highly consistent [58]. However, hand grip was tested only in sitting not in standing position. One can argue that a standing position might enable measurable co-contraction of the core. Core strength was tested with a strength endurance test. Thus, no conclusions can be made on the associations of maximal hand grip strength with maximal core strength. Further, these results do not lead to conclusions on minimal clinical important differences or thresholds of “good health”, for which an external validation and more data would be essential.

## Conclusion

Hand grip strength cannot be used as a proxy for core strength endurance in people with axSpA. Even though regular fitness assessments in people with axSpA are recommended, the choice of the best assessments for different settings is challenging.

## Electronic supplementary material

Below is the link to the electronic supplementary material.

Supplementary Material 1

## Data Availability

The data used and analyzed during the current study are available from the corresponding author on reasonable request.

## Abbreviations

axSpA: axial Spondyloarthritis
CST: Core Strength Endurance test battery
SVMB: Swiss Ankylosing Spondylitis Assoziation (Schweizerische Vereinigung Morbus Bechterew)
WHO: World Health Organisation
BASDAI: Bath Ankylosing Spondylitis Disease Activity Index
ASAS-HI: ADAS Health Index
SD: standard deviation
cm: centimeter
kg: kilogram
kgf: kilogram force
