# Supplementary figures and images for: Association between grip and core muscle strength in people with axial spondyloarthritis and healthy controls
Source: BMC Rheumatol. 2025 Oct 21;9:124. doi: 10.1186/s41927-025-00575-y (PMC12542177; doi:10.1186/s41927-025-00575-y)

Appendix A

Figure A1, Diagnostic plots


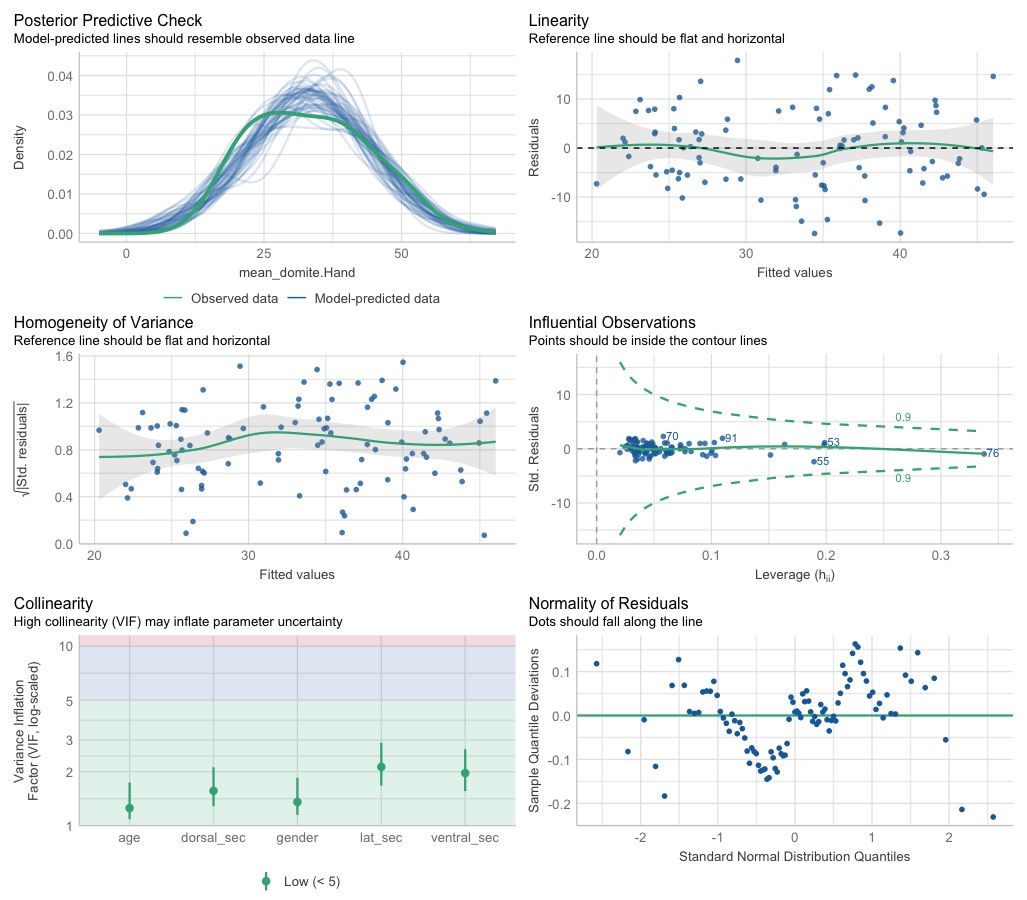

Supplement: Supplementary file 1 — Supplementary Material 1 [file 41927_2025_575_MOESM1_ESM.docx]
